# Construction Biotechnology: Integrating Bacterial Systems into Civil Engineering Practices

**DOI:** 10.3390/microorganisms13092051

**Published:** 2025-09-03

**Authors:** Olja Šovljanski, Ana Tomić, Tiana Milović, Vesna Bulatović, Aleksandra Ranitović, Dragoljub Cvetković, Siniša Markov

**Affiliations:** 1 Faculty of Technology Novi Sad, Department of Biotechnology, University of Novi Sad, Bulevar cara Lazara 1, 21000 Novi Sad, Serbia; oljasovljanski@uns.ac.rs (O.Š.); a.ranitovic@uns.ac.rs (A.R.); cveled@uns.ac.rs (D.C.); sinisam@tf.uns.ac.rs (S.M.); 2 Faculty of Technical Sciences, University of Novi Sad, Trg Dositeja Obradovića 6, 21000 Novi Sad, Serbia; tiana.milovic@uns.ac.rs

**Keywords:** construction biotechnology, microbial-induced calcium carbonate precipitation (MICP), biocementation, sustainable construction materials

## Abstract

The integration of bacterial biotechnology into construction and geotechnical practices is redefining approaches to material sustainability, infrastructure longevity, and environmental resilience. Over the past two decades, research activity in construction biotechnology has expanded rapidly, with more than 350 publications between 2000 and 2024 and a five-fold increase in annual output since 2020. Beyond bibliometric growth, technical studies have demonstrated the remarkable performance of bacterial systems: for example, microbial-induced calcium carbonate precipitation (MICP) can increase the compressive strength of treated soils by 60–70% and reduce permeability by more than 90% in field-scale trials. In concrete applications, bacterial self-healing has been shown to seal cracks up to 0.8 mm wide and improve water tightness by 70–90%. Similarly, biofilm-mediated corrosion barriers can extend the durability of reinforced steel by significantly reducing chloride ingress, while bacterial biopolymers such as xanthan gum and curdlan enhance soil cohesion and water retention in eco-grouting and erosion control. The novelty of this review lies in its interdisciplinary scope, integrating microbiological mechanisms, materials science, and engineering practice to highlight how bacterial processes can transition from laboratory models to real-world applications. By combining quantitative evidence with critical assessment of scalability, biosafety, and regulatory challenges, this paper provides a comprehensive framework that positions construction biotechnology as a transformative pathway towards low-carbon, adaptive, and resilient infrastructure systems.

## 1. Introduction

### 1.1. Motivation for Integrating Microbiology into Civil Engineering

The construction industry is undergoing a critical transformation as it seeks sustainable, cost-effective, and environmentally responsive materials and techniques. Traditional civil engineering practices rely heavily on high-carbon-footprint materials, which account for nearly 11% of global CO_2_ emissions [1]. Meanwhile, advances in bacterial biotechnology offer opportunities to revolutionise material synthesis, soil stabilisation, and structural rehabilitation through biologically driven mechanisms. Microorganisms, particularly bacteria, can facilitate the controlled transformation of material properties that would otherwise evolve slowly through natural autogenic processes, such as mineral consolidation, crack closure, or porosity reduction. This bacterial potential provides a compelling rationale for embedding living systems into the lifecycle of construction and building materials, enabling in situ repairing, enhanced durability, and reduced environmental impact [2,3].

### 1.2. Historical Context and Emerging Need

Although ancient building practices indirectly leveraged biological components, the conscious integration of microbiology into engineering design is a recent phenomenon. Over the past two decades, construction biotechnology has progressed from proof-of-concept experiments to real-world applications, particularly in geotechnical engineering and sustainable urban development [4]. The field has evolved in response to urgent environmental imperatives, including resource scarcity, urbanisation, and the structural degradation of ageing infrastructure. These challenges have fueled a search for regenerative materials and biologically adaptive solutions, making biotechnology a timely and necessary innovation for the built environment [5].

**Figure 1:**
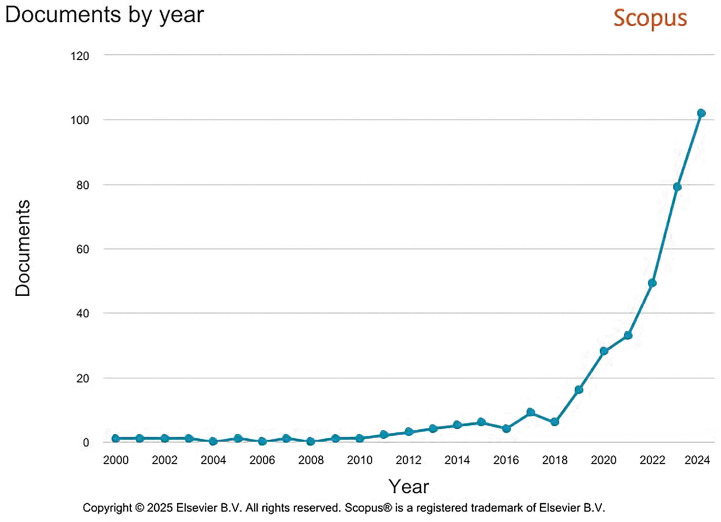
Annual distribution of documents from 2000 to 2024 based on selected keywords, retrieved from the Scopus database (accessed on 7 May 2025).

A bibliometric analysis was conducted using the Scopus database on 7 May 2025. The analysis was based on the “title-abstract-keyword” (TITLE-ABS-KEY) search function, utilising the following query terms: “construction biotechnology”, “bacterial construction materials”, “bio-based construction materials”, “engineered living materials”, “living infrastructure”, “bacterial building systems”, and “biological construction methods”. The search was limited to publications from the year 2000 to 2024 (PUBYEAR > 1999 AND PUBYEAR < 2025). The dataset includes 354 documents (223 research articles, 60 review papers, 26 book chapters, 25 conference papers, and 20 other publications). The resulting publication trends are illustrated in Figure 1, showing a notable increase in research activity, particularly over the past five years. The bibliometric trend of publications related to construction biotechnology and related themes shows a distinct temporal evolution. Between 2000 and 2015, the volume of research activity remained relatively low and stable, suggesting that the integration of biotechnology into construction practices was either in its conceptual phase or confined to a niche community of researchers. During this period, only a limited number of publications emerged annually, indicating that the field had yet to gain substantial academic traction. A modest increase began to take shape between 2016 and 2019, which may reflect the early stages of interdisciplinary interest, particularly as awareness of sustainable construction practices and climate-conscious infrastructure began to rise globally. This period likely marks the foundational development of core ideas and experimental frameworks in bacterial and bio-based construction materials. From 2020 onward, the data reveal a sharp acceleration in research output, with a steep rise in the number of documents published each year, which has continued to date. This rapid growth signals a significant shift in the field’s development, likely driven by multiple converging factors. Among these are advancements in synthetic biology, increased attention to climate resilience, and the growing feasibility of engineered living materials in practical applications. The spike in interest may also reflect increasing global investment in sustainable technologies, along with heightened interdisciplinary collaboration between microbiology, materials science, and civil engineering.

Over the past two decades, the field of construction biotechnology has evolved from conceptual studies and laboratory-scale demonstrations to more application-oriented research. Foundational studies conducted between 2000 and 2020 were instrumental in characterising microbial pathways, optimising environmental conditions for microbial activity, and developing protocols for soil improvement, crack healing, and surface consolidation. Key contributions during this period, such as the pioneering work on MICP by Whiffin [6], the first large-scale field trials led by van Paassen et al. [7], and investigations into microbial soil stabilisation by DeJong et al. [8], established experimental frameworks that continue to support current innovations in bio-mediated materials and systems. These foundational efforts laid the groundwork for the recent exponential rise in publications after 2020, as researchers increasingly explore the translation of these technologies into engineering practice. Figure 1 reflects this alteration from exploratory to implementation-focused studies, underscoring the emerging need for robust, field-validated solutions in sustainable construction.

Overall, the observed trend points to the emergence of construction biotechnology as a rapidly expanding field. The marked rise in publications after 2020 underscores its transition from a theoretical or exploratory domain into a vibrant and dynamic research area with strong potential for real-world impact [6]. If this growth continues, it may signal the onset of a new standard in infrastructure development, one where biological and engineered systems coalesce to support sustainable, adaptive, and resilient built environments.

### 1.3. Definition and Scope of Construction Biotechnology

Construction biotechnology is an emerging interdisciplinary field that integrates biological processes, particularly bacterial activity, into the design, construction, and maintenance of infrastructure [9]. It involves the strategic application of bacteria, bacterial consortia, or biologically derived materials to enhance the mechanical, environmental, and functional performance of civil engineering systems [10]. At its core, construction biotechnology leverages bacterial mechanisms such as biomineralisation in general and its specific types, such as biocementation, bioaggregation, biofilm-mediated interactions, and biopolymer production, to influence material behaviour and ground conditions. In this context, biomineralisation refers specifically to microbiologically induced mineral precipitation, typically mediated by bacterial metabolism, which leads to the formation of solid mineral phases such as calcium carbonate within or on engineered substrates [11]. These mechanisms enable the development of self-healing materials, soil stabilisers, corrosion-resistant reinforcements, and bioremediation frameworks for polluted built environments [10].

Unlike conventional construction techniques, which rely heavily on synthetic additives and energy-intensive processes, bacterial approaches offer sustainable, adaptive, and often self-regulating alternatives [10]. This microbial approach offers a promising, eco-compatible alternative to traditional chemical-based soil and material stabilisation methods, with expanding relevance in both laboratory research and field-scale applications. Studies from various international groups have demonstrated the effectiveness of bacterial-mediated processes in improving soil strength, reducing permeability, and enabling crack repair in concrete and masonry systems [12,13,14]. The integration of bacterial functions into infrastructure contributes to reduced carbon emissions, enhanced durability, and resilience to environmental stressors, aligning with broader goals in sustainable development, green building, and climate-adaptive engineering [15,16]. The construction biotechnology scope spans a wide array of applications, including geotechnical engineering (i.e., biogrouting, slope stabilisation, and subgrade improvement), civil engineering (self-healing concrete, bacterial surface hardening, and crack repair), environmental engineering (bacterial bioremediation, contaminant immobilisation, and CO_2_ sequestration), materials science (development of bio-admixtures, encapsulated bacterial agents, and engineered living materials) [10,17,18], etc.

**Figure 2:**
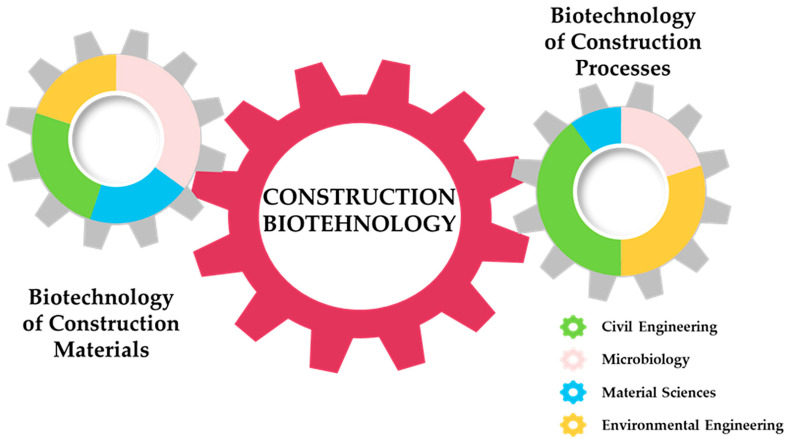
Conceptual framework of construction biotechnology represented through two interlocking gears (the colour-coded segments within each gear (green, pink, blue, and yellow) correspond to key thematic areas in the field). The relative proportions of these segments are illustrative and intended to provide a visual impression of the balance and interconnection among different research directions within construction biotechnology. This conceptual model emphasises the interdisciplinary structure and integrated development of the field.

Figure 2 provides a conceptual model of construction biotechnology, presented as two interlocking gears to symbolise the dynamic interplay between its two core domains: materials and processes. The left gear highlights materials-related applications, such as bio-concrete, microbial mortars, and biopolymer-enhanced structures, while the right gear illustrates process-oriented approaches, including biocementation, biocleaning, biosealing, and bioremediation. Each segment within the gears is colour-coded (green, pink, blue, and yellow) to represent distinct thematic categories within the field. This integrative representation helps contextualise the breadth of construction biotechnology, emphasising that both material innovations and biological processes are essential and interconnected components of its advancement. Comparable interdisciplinary frameworks have been used in previous studies to visualise complex, emerging fields [8,12].

The evolution of construction biotechnology has been marked by a growing interest in microbial mechanisms that go beyond classical biomineralisation. Recent studies have increasingly focused on pathways such as biofilm-mediated consolidation, enzymatic precipitation, and microbial biopolymers, which offer complementary or alternative approaches to MICP [12,13]. Additionally, practical implementation frameworks and pilot-scale trials have demonstrated the feasibility of these approaches in diverse geotechnical contexts [14]. These advancements suggest that the field is undergoing a transition from narrowly focused laboratory investigations to broader applications that leverage diverse microbial processes for eco-efficient construction. It can be assumed that, as the field continues to evolve, it is increasingly incorporating advances from synthetic biology, genetic engineering, and computational modelling to create programmable, responsive, and digitally integrated biological systems [19,20]. This convergence marks a model shift: from treating infrastructure as inert assemblies to designing it as bio-integrated, living systems capable of interaction, adaptation, and regeneration.

## 2. Bacterial Contribution and Mechanisms Relevant to Civil Engineering

Construction biotechnology leverages the biochemical versatility of bacteria to manipulate material properties, facilitate self-repair, and enhance environmental performance in infrastructure systems [21]. Within construction biotechnology and biogeotechnics, microbially induced carbonate precipitation (MICP) has received the most research attention to date, though other bacterial processes are gaining relevance [22]. This section provides a detailed overview of the core and auxiliary bacterial mechanisms that support biological interventions in the civil engineering industry.

**Figure 3:**
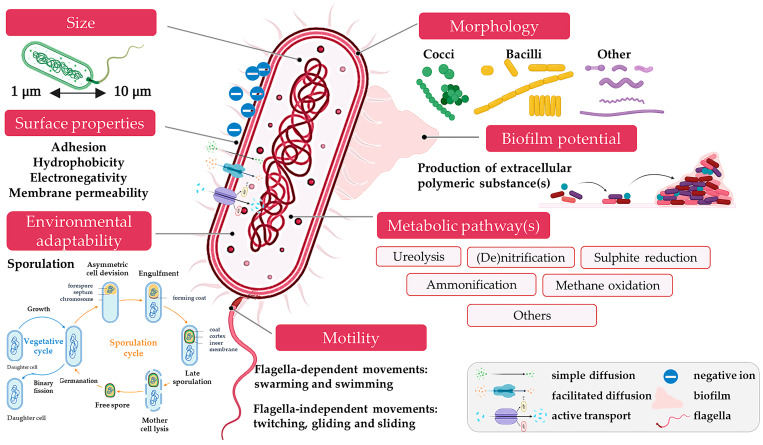
The potential of individual bacterial cells in construction materials and processes.

First and foremost, each active bacterial cell can contribute to the construction material or process as a multifunctional mediator. Figure 3 visualises the structural and functional attributes of bacterial cells that reinforce their unique potential in transforming civil engineering practices through bio-mediated processes. Bacteria display a wide range of cell sizes and morphologies, typically between 1 and 10 µm. Their physical dimensions determine their mobility through porous construction substrates, colonisation efficiency, and attachment potential. For instance, rod-shaped bacteria often exhibit enhanced adhesion to mineral surfaces and are commonly employed in crack-healing applications [23]. Morphological plasticity, such as filamentous growth or cell chaining, also supports biofilm development under stress, aiding long-term structural integration [24,25]. At the core of bacterial-material interactions lies the bacterial cell envelope, which mediates adhesion, ion exchange, and signal sensing. Bacteria utilise simple diffusion for passive nutrient absorption and facilitated diffusion for specific ions such as Ca^2+^, as well as active transport mechanisms for controlling intracellular ion balance in harsh environments, including those found in concrete pores. These systems are crucial when bacteria induce the MICP process, as they require controlled ion flow and pH shifts [26]. Moreover, surface hydrophobicity and charge influence how bacteria anchor to sand grains or steel, affecting the success of bio-mediated soil stabilisation and corrosion protection [27].

One of the most potent bacterial tools is the ability to form biofilms—structured microbial communities embedded in a matrix of extracellular polymeric substances (EPS). In the construction context, biofilms act as adhesive agents that secure bacteria within cracks, create localised microenvironments for mineral precipitation, and form protective coatings on steel to prevent oxygen and ion ingress, mitigating corrosion. Biofilms are essentially living construction aids, self-regulating and responsive to environmental cues [28]. Their role is particularly vital in underwater or chloride-rich environments where conventional sealants fail [29]. On the other hand, bacterial-mediated biofilms are also associated with material degradation processes such as biocorrosion in concrete, plumbing, and metallic reinforcements [30,31,32].

Bacteria possess an incredible range of metabolic pathways that allow them to function as micro-scale construction chemists. Microbial mechanisms behind construction biotechnology encompass both core and supporting biochemical pathways. Among the core mechanisms, ureolysis is the most widely studied and applied due to its direct role in calcium carbonate precipitation under alkaline conditions. Denitrification and sulphate reduction are also relevant, particularly when used to induce carbonate precipitation or to sequester heavy metals in contaminated construction matrices. While methane oxidation and ammonification are not directly involved in structural enhancement, they have been investigated in niche applications such as microbial gas control in anaerobic foundations or pollutant transformation in soil-cement composites. These auxiliary mechanisms contribute to the broader field only when coupled with biomineralisation processes or targeted bioremediation strategies relevant to construction materials [33]. Among the mentioned metabolic pathways, ureolysis remains the most direct and widely utilised for biomineralisation in construction applications, although denitrification has also been explored under specific engineered conditions.

What truly sets bacteria apart is their resilience. They can function in alkaline pH, low oxygen, saline conditions, or even contaminated soils, making them ideal for long-term deployment in real-world built environments. Some strains become dormant under dry conditions and reactivate upon wetting, allowing for timed biological responses, such as crack healing, when water infiltrates concrete [34]. This adaptability also means bacteria can thrive in locations ranging from offshore platforms to desert pavements, offering unmatched sustainability and flexibility in materials engineering. Finally, motility, whether through flagella, twitching pili, or chemotaxis, enables bacteria to explore and colonise complex environments, such as microcracks, soil matrices, or pore networks. This movement ensures the effective delivery of bacterial action where it is needed most, whether initiating healing at the tip of a crack or migrating into loose sediments for stabilisation [35]. Motile bacteria are often better suited for in situ applications, as they can self-disperse without the need for mechanical mixing or injection pressure, thereby reducing engineering costs and effort [36]. Bacterial motility contributes to dispersion at the microscale, but effective delivery in construction systems often requires external mechanisms such as injection, percolation, or infiltration and is highly influenced by matrix properties like porosity and permeability [22].

Bacteria are not just passive agents in construction biotechnology, but they are responsive, dynamic, and self-organising systems. Their small size hides enormous biochemical potential [37]. Engineering approaches used in this context include optimising behaviours through strain selection, adaptation, or stimulation strategies such as nutrient enrichment. The potential of bacterial systems makes them more innovative, more sustainable, and more aligned with the ecological challenges of our time.

### 2.1. Microbial-Induced Carbonate Precipitation

**Figure 4:**
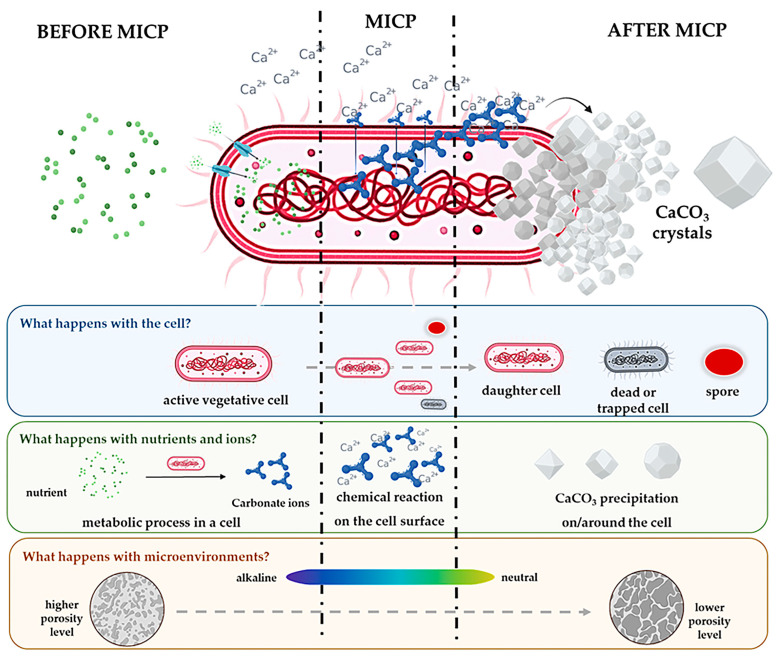
The MICP process mediated by bacteria.

MICP is a biomediated process in which specific bacteria induce the formation of calcium carbonate (CaCO_3_) crystals through multifunctional roles (Figure 4). *Sporosarcina pasteurii* is the most widely studied bacterium for this application, as it produces urease, which catalyses urea into ammonium and carbonate ions. These ions then react with calcium ions to precipitate carbonate [38,39]. This reaction forms a matrix that binds soil particles or heals cracks in concrete, enhancing the structural integrity of materials. MICP has been successfully applied in self-healing concrete, erosion control, and geotechnical engineering, offering significant advantages in terms of sustainability and energy efficiency. Its repeatability and potential for field-scale deployment have been confirmed in multiple large-scale experiments [40,41]. Field-scale applications of MICP have advanced significantly over the past decade, demonstrating the potential for bio-based technologies to be translated from laboratory-scale innovation to real-world geotechnical solutions. Several successful implementations have validated the scalability and technical feasibility of MICP for soil stabilisation, erosion control, and crack remediation in diverse soil and climatic conditions. Notably, some researchers reported robust outcomes from large-scale trials, including improved shear strength and reduction of hydraulic conductivity in treated soils [6,7]. DeJong et al. [42] further confirmed that microbially induced cementation could be reliably induced in situ through controlled injections and environmental conditioning, with performance metrics closely tied to site-specific parameters such as injection strategy, soil grain size, and microbial activity.

Figure 4 presents a schematic overview of the MICP process, emphasising the sequential stages of urea hydrolysis, carbonate ion generation, and calcium carbonate precipitation. While simplified for visual clarity, the diagram summarises complex microscale dynamics, including substrate porosity, pH gradients, ionic strength, and the distribution of microbial biomass. It is important to note that carbonate precipitation commonly occurs both on microbial surfaces and in the interstitial spaces between soil particles, depending on environmental and chemical conditions. Additionally, the designation “active vegetative cell” is used to indicate a metabolically functional state and does not preclude facultative or diverse physiological capabilities typical of ureolytic bacteria.

In Figure 4, each horizontal panel corresponds to a core element of MICP: cell status, ion/nutrient transformation, and porosity changes in the microenvironment. These interactions highlight the intricately coordinated role that bacterial cells play as catalysts for biomineralisation in construction biotechnology [43]. Observing bacterial cells before MICP, bacteria such as *S. pasteurii* exist in an active vegetative state, metabolically functional and capable of urea hydrolysis. These cells are introduced or reactivated upon exposure to moisture or specific nutrients. During MICP, as the cells hydrolyse urea (or alternative nitrogen sources), they begin to divide, forming daughter cells. Some may also begin to transition to dormant or trapped states as mineral layers form around them [44]. After MICP, cells that become encased in precipitated CaCO_3_ may either die or sporulate, depending on environmental conditions such as oxygen, pH, and nutrient availability [45]. Spore formation is a crucial survival mechanism that allows long-term reactivation under favourable conditions [46,47]. In terms of nutrients and ions in the system, nutrients such as urea, calcium, and carbon sources diffuse into the microenvironment in the input stage. The urease enzyme catalyses the hydrolysis of urea into ammonia and carbon dioxide, which subsequently equilibrate in solution to form carbonate and bicarbonate ions under alkaline conditions. In the reaction phase, these carbonate ions then react with calcium ions (Ca^2+^) present in the system. This interaction, mostly occurring on the cell surface or within the EPS layer, leads to localised supersaturation. As a result, CaCO_3_ crystals nucleate and grow on and around bacterial cells. The morphology and distribution of these crystals affect both material integrity and permeability [28].

Furthermore, the microenvironment undergoes numerous changes. Before MICP, the environment is typically porous, with high permeability and low structural cohesion. The presence of nutrients maintains bacterial viability. During MICP, as metabolic activity proceeds, localised alkalinity increases (pH ~9). This pH shift is critical to promoting CaCO_3_ precipitation. After MICP, the accumulation of carbonate crystals significantly reduces porosity and permeability. This solidification enhances material performance for applications such as crack sealing, surface hardening, and soil stabilisation [48]. Figure 4 underscores the dynamic and multifunctional nature of bacterial cells in the MICP process. Not only do they act as biocatalysts, but their cellular lifecycle and metabolic products also actively reshape the surrounding environment. The transformation from a biologically active zone to a mineralised matrix reveals the self-regulating potential of bacterial systems in structural healing and durability enhancement. This biomineralisation cycle is not merely a chemical process, but it is an engineered bacterial lifecycle that bonds microbiology, material science, and geotechnical engineering. Its implications extend from self-healing concrete and biogrouting to eco-cementation and restoration of heritage structures [49].

In general, MICP has been extensively studied using ureolytic bacteria such as *Sporosarcina pasteurii* and *Bacillus sphaericus*, which exhibit high urease activity and are capable of inducing robust carbonate crystallisation. Šovljanski et al. [33] reviewed a wide range of natural isolates from sediments, soils, cement-related sources, and references from different collections of microorganisms, as well as genetically modified bacteria that are used in engineered MICP processes. The optimal environmental conditions for effective MICP typically include a temperature range of 25–35 °C, pH levels between 8.0 and 9.5, and calcium ion concentrations of 0.1–0.5 M, which facilitate carbonate supersaturation and crystal nucleation on bacterial surfaces or extracellular polymeric substances [25,50]. Quantitatively, studies have reported significant improvements in mechanical properties following MICP treatment. For example, one research group demonstrated that the compressive strength of sand columns treated with *S. pasteurii* increased from 80 kPa (untreated) to over 1000 kPa post-treatment, depending on the number of treatment cycles and injection concentration [42]. Similarly, the use of MICP in biogrouting has led to enhancements in shear strength by 60–70% and reductions in permeability by over 90% in field-scale trials [12,51]. Additionally, MICP refers to the biologically mediated formation of CaCO_3_ through various bacterial metabolic activities. Together, these bacterial mechanisms represent a diverse and adaptable toolkit for biogenic mineralisation in civil engineering contexts [33]. Their comparative attributes—ranging from bacterial types to environmental requirements and application domains—are summarised in Table 1. Among the best-studied is ureolysis-induced carbonate precipitation, which involves the enzymatic hydrolysis of urea by ureolytic bacteria such as *Sporosarcina* and *Bacillus* species. This process produces carbonate ions that combine with calcium in the environment to form CaCO_3_, resulting in a biomineral matrix that strengthens granular media, seals cracks, and enhances material durability [28,42]. Ureolysis-driven MICP has been effectively deployed in soil stabilisation, self-healing concrete, and restoration of porous stone and masonry. However, its application faces challenges, including ammonia release and uneven carbonate deposition, particularly in large or heterogeneous substrates [52]. Beyond ureolysis, alternative carbonate-precipitating pathways are gaining attention due to their environmental or contextual suitability. For example, denitrification-induced carbonate precipitation uses facultative anaerobic bacteria (e.g., *Pseudomonas denitrificans*, *Paracoccus* spp.) to reduce nitrate to nitrogen gas while producing carbonate ions under anoxic conditions. This makes it ideal for deep foundations and saturated soils where oxygen is limited, while also mitigating the ammonia-related drawbacks of ureolysis [38].

Similarly, sulphate reduction—a pathway utilised by sulphate-reducing bacteria such as *Desulfovibrio* spp.—generates hydrogen sulphide that reacts with divalent metal ions (e.g., Fe^2+^, Zn^2+^) to form insoluble metal sulphides. These reactions are particularly valuable for immobilising heavy metals in contaminated construction sites and reducing corrosion in subsurface pipelines or foundations [53]. A further distinct mechanism is photosynthetic carbonate precipitation, which relies on the metabolic activity of cyanobacteria (e.g., *Synechococcus*, *Gloeocapsa*) to consume atmospheric CO_2_ and increase local pH, thereby promoting carbonate precipitation in light-exposed environments. This bio-induced mineralisation contributes to carbon sequestration and the development of bio-integrated façade systems or carbon-negative building envelopes [54].

However, MICP is not a single, uniform process. Instead, it encompasses a variety of sub-processes and related mechanisms that differ in their biochemical pathways, material interactions, and functional outcomes. Namely, these bacterial-induced processes share the overarching goal of enhancing material performance via bacterial activity, but they differ significantly in their underlying mechanisms, material compatibility, and engineering outcomes. As summarised in Table 2, three principal MICP-related techniques, bioconsolidation, biocementation, and bioaggregation/biogrouting, are compared in terms of definition, bacterial contribution, and applications. These processes share the common objective of improving material performance through bacterial intervention, but they diverge significantly in terms of mechanisms, applications, and engineering benefits [33]. Bioconsolidation refers to bacterial-induced mineral precipitation that fills voids and pores within porous substrates such as sandstone, historical masonry, or cracked concrete. This process typically relies on in situ CaCO_3_ deposition by strains such *as S. pasteurii* and *Bacillus* spp., enhancing surface cohesion and reducing permeability. Given its minimally invasive nature, it is especially suitable for heritage conservation and passive crack sealing where structural integrity must be preserved without heavy machinery or chemical intervention [55].

Biocementation, in contrast, focuses on strengthening granular soils (e.g., sands or silts) through the targeted deposition of minerals, typically calcium carbonate, at interparticle contacts. This method enhances shear strength, stiffness, and erosion resistance, making it highly effective for soil stabilisation, liquefaction mitigation, and subgrade reinforcement. It is often considered the biological analogue to chemical grouting and is sensitive to environmental factors such as pH, flow rates, and saturation. Ureolytic bacteria like *S. pasteurii* are commonly employed, though denitrifying bacteria (*Pseudomonas denitrificans*, *Paracoccus* spp.) have also shown promise, particularly in anaerobic zones using nitrate-based pathways [56].

Bioaggregation (also referred to as biogrouting in some literature) involves bacterial secretion of extracellular polymeric substances (EPS) that function as organic adhesives, enhancing cohesion among fine-grained or organic-rich soils. This approach is less reliant on mineralisation and is more adaptable to clayey soils, sludge, or erosion-prone areas. Bacterial strains such as *Pseudomonas* spp., *Azotobacter* spp., and *B. subtilis* are used to promote flocculation, water retention, and slope stability. This method supports vegetative root systems and biological crust formation, with advantages in moisture retention and ecological restoration [51,57]. Biogrouting applications benefit particularly from MICP due to the targeted deposition of CaCO_3_ at grain contacts in granular soils. These processes increase the stiffness and cohesion of treated layers but also provide long-term durability under fluctuating environmental conditions. *P. denitrificans* and *Paracoccus* spp. have also been employed in denitrification-driven MICP for anaerobic zones, offering an environmentally friendlier alternative to urea-based methods while still achieving comparable mechanical improvements [39,43].

### 2.2. Biocleaning

**Figure 5:**
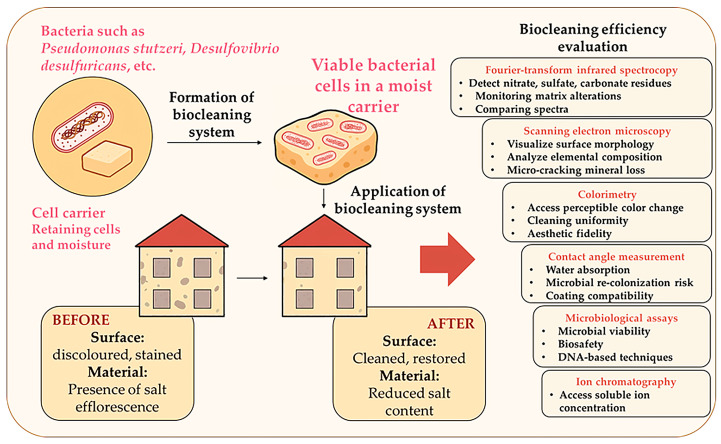
Biocleaning of the material surface using specific bacteria.

Biocleaning represents a sustainable biotechnological method that employs viable bacterial cells or their enzymatic systems (Figure 5) to selectively remove unwanted surface deposits, such as salt efflorescence, nitrate crusts, black crusts, and organic residues, from construction and heritage materials without compromising substrate integrity [58,59]. The technique is rooted in the natural ubiquity and environmental adaptability of bacteria, particularly non-pathogenic strains such as *Pseudomonas stutzeri* and *Desulfovibrio desulfuricans*, as well as extremophiles from salt-tolerant genera. These bacteria are capable of colonising building substrates in varying environmental contexts and metabolising specific compounds to promote localised cleaning [60]. Initial studies in the late 1990s demonstrated the potential of bacterial action in reducing salt crystallisation phenomena, which are a significant threat to porous building materials in historical structures [61]. The term “biocleaning” was thus coined to emphasise the biological origin of the cleaning mechanism, setting it apart from conventional chemical or abrasive treatments [58].

Biocleaning operates through one or more of the following mechanisms:

- Denitrification: Bacteria such as *P. stutzeri* reduce nitrates that form efflorescence on frescos and limestone façades.
- Desulfation: Sulphate-reducing bacteria (SRB) remove gypsum or black crusts resulting from atmospheric pollution.
- Enzymatic biodegradation: Specific bacterial enzymes catalyse the breakdown of binders, adhesives, and organic films on painted surfaces.

As presented in Figure 5, the delivery system plays a pivotal role in maintaining bacterial viability. Gel-based matrices (e.g., agar, gellan gum, or Carbogel) are used to immobilise bacterial cells, maintain hydration, and facilitate even application across complex geometries, including vertical or highly textured surfaces [62]. An ideal carrier should retain viable cells and moisture for extended periods, adhere to various surfaces (both rough and smooth, as well as vertical and horizontal), and be easy to apply and remove after treatment. Despite its high selectivity and ecological compatibility, several critical considerations must be addressed before implementing it in the field. For example, pathogenicity screening is mandatory to ensure safety. The efficacy of bacteria should be validated through laboratory-scale tests. At the same time, interaction with the substrate must be examined to avoid unintended degradation or discolouration. Additionally, carrier-substrate compatibility is crucial to maintain microecological balance during treatment [63].

In contrast to classical applications in soil or water bioremediation, bacterial strains used for biocleaning often operate under nutrient-limited, stress-prone conditions. As such, short activity windows and viability loss can occur, emphasising the importance of optimising cell carriers and environmental parameters. Numerous studies have demonstrated the success of biocleaning, as presented in Table 3. The most frequently targeted contaminants include nitrate efflorescence, sulphated black crusts, and mixed salt pollutants. *P. stutzeri* has been repeatedly employed for the removal of nitrate and organic contaminants, consistent with its known metabolic capabilities and safety profiles. Anaerobic sulphate-reducing bacteria such as *D. desulfuricans* were successfully utilised for the selective removal of gypsum-based crusts, while halotolerant *Bacillus* spp. demonstrated potential for broader pollutant profiles, particularly in outdoor environments [64].

The application methods and carrier systems varied significantly depending on the substrate type and operational requirements. Carbogel- and agar-based hydrogels were primarily used for localised, controlled treatments on stone and frescoes. In contrast, dry gels with moisture activation were introduced for scalable interventions on porous brick walls. All studies reported successful removal of undesirable layers without causing morphological or chemical damage to the underlying material. Additionally, the biocleaning agents were selected to be non-pathogenic and environmentally safe, supporting their use in the conservation of various building materials. These findings underline the importance of tailoring bacterial treatments to specific conservation challenges. Factors such as substrate porosity, pollutant composition, environmental exposure, and the scale of required intervention must be carefully considered when designing a biocleaning strategy. Therefore, an extensive spectrum of methods for evaluating biocleaning efficiency, such as FTIR and SEM analysis, colourimetry, ion chromatography, contact angle measurement, and microbiological testing, should be employed (Figure 5).

While biocleaning remains a relatively young methodology, its growing validation through field trials and controlled studies positions it as a promising alternative in sustainable heritage conservation and the restoration of eco-conscious materials. Unlike the classic applications of denitrifiers in the bioremediation processes of soil and water ecosystems, the use of these bacteria in the field of biocleaning of cultural heritage materials is accompanied by numerous, insufficiently studied specificities, such as (1) the impossibility of introducing nutrient media for cell multiplication, which directly affects the short and limited activity of the strains; (2) the problem of maintaining cell viability and vitality; (3) the influence of the surface characteristics of the material that needs to be treated and the bacteria carrier [65]. Although the biocleaning process is relatively new in the field of protecting cultural heritage materials, numerous studies confirm its high efficiency in removing salt and other deposits from various materials at the laboratory level or in limited areas of facilities (Table 3). However, for the needs of biocleaning on a larger scale (e.g., complete objects of cultural heritage), it is necessary to produce enough bacterial biomass [66].

### 2.3. Biofilm Formation and Material Interactions

**Figure 6:**
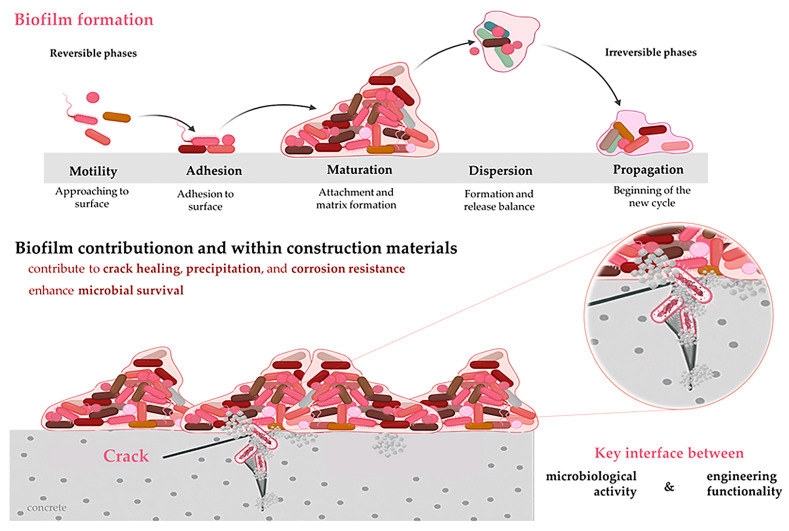
Biofilm contribution to the engineering practice.

Biofilms, aggregated microbial communities enclosed within a self-produced matrix of extracellular polymeric substances (EPS), play a foundational role in the efficacy and durability of bacterial systems in construction biotechnology. As shown in Figure 6, their formation on and within materials represents a key interface between biological activity and engineering functionality [67,68]. In the context of concrete, soils, and composites, biofilms not only enhance bacterial survival but also actively contribute to crack healing, carbonate precipitation, and corrosion resistance. In engineered environments, biofilms serve both as biological anchoring systems and biochemical interfaces between bacterial processes and structural materials [69]. These bacterial matrices enhance the localisation and persistence of metabolic activity, promoting targeted carbonate precipitation and contributing to crack healing and corrosion mitigation. Importantly, biofilms act as diffusion barriers, protecting embedded bacteria from extreme pH levels, drying, and other environmental stresses that occur daily in concrete or subsurface environments [70]. The dynamics of biofilm formation—governed by substrate surface chemistry, hydrodynamic flow, and bacterial community composition—determine their mechanical integrity and mineralisation efficacy [50]. Biofilm-based systems have been successfully deployed in self-healing concrete, where encapsulated bacteria become activated upon crack formation and precipitate CaCO_3_ within the biofilm matrix, thereby restoring structural continuity [71]. Biofilm formation allows bacterial cells to adhere to the surfaces of cementitious matrices, particularly in microcracks or voids, where they remain protected from desiccation, alkaline pH, and mechanical stress [72]. The adhesive EPS matrix functions as both a protective barrier and a reaction site for biomineralisation, supporting sustained bacterial activity even under harsh construction conditions [73].

In self-healing concrete, biofilms contribute to the localised nucleation and growth of CaCO_3_ crystals. When cracks occur, water ingress activates dormant or encapsulated bacterial spores. These bacteria, upon reactivation, begin to form biofilms within the cracks and subsequently initiate MICP (Figure 6), effectively sealing the cracks [74]. This localised biofilm-mediated calcification has been shown to improve both the strength and impermeability of repaired concrete sections [75]. The hydrophobic and electrostatic properties of biofilms also influence their interaction with metallic components embedded in concrete. Several studies suggest that biofilms reduce corrosion by acting as oxygen diffusion barriers, thereby inhibiting the cathodic reaction that drives the oxidation of rebar [76]. Moreover, biofilms can selectively interact with ions and organic molecules, contributing to surface passivation and chemical stabilisation [28]. Recent advances in synthetic biology and bacterial encapsulation techniques have enabled the tuning of biofilm characteristics, including thickness, adhesive strength, and mineral affinity, for targeted engineering outcomes. For instance, bacteria engineered to overproduce EPS can form thicker, more resilient biofilms, which are especially valuable in crack healing and slope stabilisation [77]. Additionally, substrate selection—such as incorporating porous carriers or mineral-rich aggregates—can enhance biofilm establishment and longevity [78].

### 2.4. Biopolymer Production

**Figure 7:**
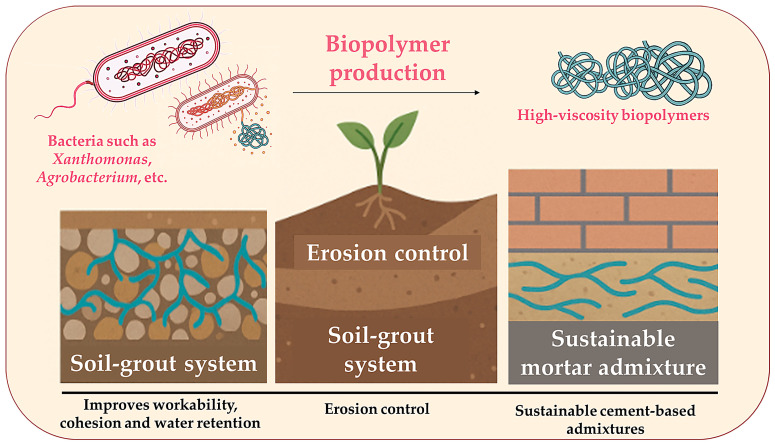
Bacterial contribution through polymer production.

In addition to inorganic mineralisation, microorganisms can synthesise biopolymers that alter the rheological and mechanical properties of soils and binders (Figure 7). Species such as *Xanthomonas campestris* and *Agrobacterium* spp. produce high-viscosity exopolymers like xanthan gum and curdlan, which improve workability, cohesion, and water retention capacity of soil-grout systems. These biogenic polymers offer environmentally benign alternatives to synthetic additives and are increasingly being considered in applications such as eco-grouting, erosion control, and sustainable cement-based admixtures [79].

Polymers such as xanthan, alginate, curdlan, dextran and gellan are typically synthesised during bacterial fermentation processes and can be triggered under stress or nutrient-limited conditions [80]. As shown in Table 4, their physicochemical characteristics, such as high viscosity, thermal stability, and gelation ability, enable a wide range of engineering functions, including soil cohesion enhancement, water retention, crack sealing, and biocementation [81,82,83,84,85].

Moreover, these biopolymers offer an environmentally friendly alternative to synthetic additives, aligning with the growing demand for sustainable materials in geotechnical and construction applications. Unlike many synthetic additives, which may persist in the environment or raise concerns about toxicity, bacterial polymers are inherently biodegradable. They can be produced through low-energy fermentation processes using renewable substrates. This not only reduces their environmental footprint but also aligns with the principles of a circular economy. Furthermore, biopolymers tend to exhibit better compatibility with natural soils and cement matrix, often improving cohesion and water retention without disrupting the surrounding ecosystem [86]. Therefore, understanding the bacterial pathways and production dynamics of these biopolymers is crucial for optimising their integration into practical engineering systems.

### 2.5. Corrosion Inhibition via Biofilm Barriers

**Figure 8:**
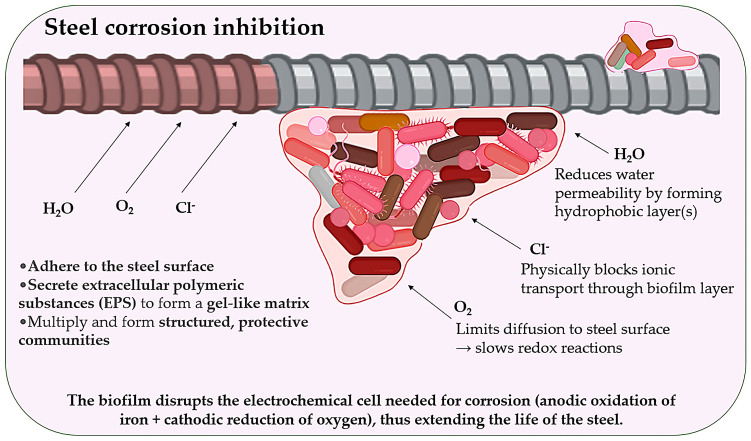
Steel surface corrosion inhibition.

Corrosion of steel reinforcement is one of the most persistent threats to the long-term durability of reinforced concrete structures, particularly in marine, coastal, or environments exposed to de-icing salts. Traditional methods for corrosion prevention often rely on chemical inhibitors, which can be costly, toxic, or environmentally unsustainable [87]. Recent advances in microbiologically influenced protection strategies (Figure 8) offer an alternative: the use of bacterial biofilms as biological corrosion barriers [88]. Particular bacterial species, including *Pseudomonas* and *Bacillus* spp., have demonstrated the ability to colonise steel surfaces and form structured biofilm layers enriched with extracellular polymeric substances (EPS) [89].

These bacterial biofilms perform several key protective functions. They adhere to the steel surface, forming a stable microenvironment, but also secrete EPS, creating a gel-like hydrophobic matrix that reduces water permeability. Additionally, biofilms physically block chloride ions and limit oxygen diffusion, both of which are key drivers of electrochemical corrosion. By interfering with the electrochemical cell required for steel corrosion, specifically the anodic oxidation of iron and cathodic reduction of oxygen, these biofilms disrupt redox reactions and significantly slow the corrosion process. Unlike synthetic inhibitors, bacterial biofilms are self-replicating, self-repairing, and biodegradable. Their use is especially promising for underwater infrastructure, sewer systems, and marine structures, where maintaining consistent protection is difficult through traditional coatings. This biological passivation strategy aligns with the principles of sustainable construction by reducing chemical inputs, leveraging natural processes, and extending the service life of structural components in corrosive environments [89,90,91,92,93,94].

### 2.6. Genetically Engineered Bacterial Systems

The frontier of construction biotechnology lies in the rational design and programming of bacterial systems through the application of genetic engineering. By leveraging genetic circuits, synthetic promoters, and quorum-sensing elements, bacteria can be engineered to detect environmental signals, such as moisture, pH shifts, or mechanical stress, and initiate targeted responses, including carbonate precipitation, polymer secretion, or the release of healing compounds [95]. These engineered living systems enable the development of adaptive, intelligent infrastructure with self-regulation capabilities [96,97]. Applications include biosensing concrete panels, stress-responsive joint sealants, and real-time degradation monitors. When integrated with digital and AI modelling tools, such bacterial systems form the basis for smart bio-integrated infrastructure, transforming passive materials into dynamic, self-regulating elements within the built environment [98].

## 3. Engineering Integration Strategies

**Figure 9:**
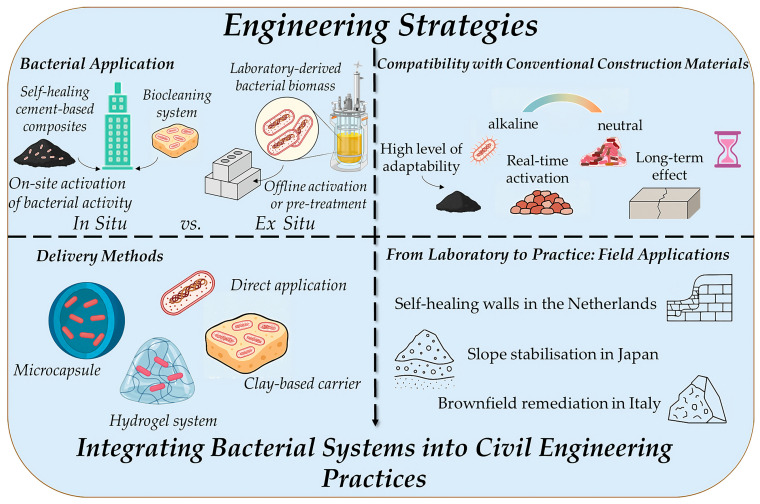
Current Engineering Strategies in Construction Biotechnology.

As construction biotechnology evolves from laboratory innovation into large-scale application, one of the biggest engineering challenges lies in effectively embedding bacterial systems into civil infrastructure [99]. This section examines how bacteria are delivered, activated, and sustained in complex built environments, including those found in soil, concrete, and contaminated sites. With recent progress in bioengineering and material compatibility, bacterial contributions are now entering the mainstream of construction as scalable, responsive technologies (Figure 9).

### 3.1. In Situ vs. Ex Situ Bacterial Application

Bacterial systems can be applied in situ (directly at the construction site) or ex situ (off-site preparation before deployment) [100]. In situ techniques are increasingly used for real-time soil stabilisation and in situ underground remediation. In this approach, bacteria are introduced or stimulated directly within the target environment. This approach is favoured for geotechnical applications, such as biogrouting and soil stabilisation, where bacterial activity must be spatially integrated within the ground matrix [38,101]. In situ biocementation has also been successfully trialled in pilot-scale experiments to stabilise slopes and foundations [102]. For example, MICP has been deployed in sandy soils to enhance resistance to liquefaction and erosion. These applications demonstrate how living systems can perform functional tasks where mechanical or chemical methods are insufficient [47,103]. Ex situ systems, on the other hand, involve cultivating bacteria in controlled settings, such as bioreactors or batch tanks, and applying them later as part of pre-treated construction materials. This method provides higher control over bacterial growth conditions and is ideal for modular building units, precast concrete panels, or bio-enhanced mortars [104,105]. Choosing between in situ and ex situ *methods often depends on environmental constraints*, *the* target application (e.g., soils vs. concrete), and the desired degree of bacterial control.

### 3.2. Delivery Methods for Achieving Timed Bacterial Response

One of the key engineering challenges in bacterial construction biotechnology is maintaining bacterial viability and activating the bacterial response precisely when and where it is needed. Environmental stressors, such as extreme pH levels, desiccation, and nutrient deficiencies, can rapidly reduce bacterial activity, particularly within harsh matrices like concrete [77]. To overcome these challenges, researchers have developed a range of encapsulation and delivery systems that protect bacteria during incorporation and enable responsive activation under specific environmental conditions. These systems are inspired by nature but designed for engineering reliability and efficiency. Recent advances include:

- Hydrogels and smart polymers that degrade or swell in response to pH shifts, moisture ingress, or mechanical damage, enabling timed or site-specific nutrient and bacterial release [106]. These materials form a responsive membrane that supports bacterial viability and metabolic activation when cracks form or water enters the system.
- Expanded clays and lightweight aggregates, which serve as bacterial shelters within concrete. Their porous internal structure allows bacteria to remain dormant for extended periods while shielding them from high alkalinity and mechanical stress [107]. Upon exposure to moisture or oxygen, bacterial activation can proceed.
- Microcapsules engineered with biofilm-inducing triggers, such as surface charge variation, temperature cues, or quorum-sensing mechanisms, help ensure that bacterial activity is initiated only in critical zones, such as crack interfaces or porous microenvironments [103].

These delivery platforms are part of a larger trend in innovative construction materials, where infrastructure is designed to respond autonomously to changes in environmental or mechanical state. The integration of materials science and microbiology enables self-healing, self-sealing, and self-monitoring capabilities in next-generation construction materials [108,109]. Interestingly, some research groups have opted for non-encapsulated bacterial systems, relying on the inherent robustness and environmental tolerance of selected bacterial strains [37]. These methods are particularly suited for environments where bacterial survivability is less of a concern, such as soil applications or short-term biogrouting processes. For instance, direct injection of bacterial suspensions into sand columns has been used successfully without encapsulation, provided that hydration and nutrient levels are carefully managed [38,46]. Additionally, the self-healing capacity of hardened cement cubes by utilising only lyophilised *Bacillus* biomass was defined in actual studies as a simplified solution with a strong background in selecting the best bacteria for engineering use [37]. Both encapsulated and unencapsulated approaches reflect a growing toolkit for bacterial delivery in construction, offering engineers flexible solutions tailored to site-specific demands.

### 3.3. Compatibility with Conventional Construction Materials

Integrating bacterial systems into concrete or soil in construction sites is not just about biology; it is also about engineering compatibility [46]. Construction materials present unique stressors for living organisms, including alkaline environments (pH > 11), oxygen and nutrient diffusion limitations, potential interference from supplementary cementitious materials (e.g., slag, silica fume), etc. However, recent studies show that bacteria such as *Bacillus sphaericus* and *S. pasteurii* remain viable and active in these environments when added or appropriately encapsulated. Moreover, MICP processes do not compromise concrete strength; instead, they may improve it by reducing permeability and microcrack propagation [104,110,111,112].

### 3.4. From Laboratory to Practice: Field Applications

The transition of bacterial technologies from laboratory-scale research to real-world civil engineering applications represents a critical milestone in the maturation of construction biotechnology [17]. Field case studies serve not only to validate the feasibility of bacterial systems under complex environmental conditions but also to identify the technical, economic, and regulatory factors that influence scalability and adoption. Several pilot and full-scale deployments worldwide illustrate the diverse potential and practical challenges associated with implementing bacterial processes in infrastructure development [101,113,114,115,116]. Collectively, these case studies underscore the versatility of bacterial systems across geotechnical, structural, and environmental domains. They also reveal several critical insights: bacterial viability and adaptability are highly context-dependent; delivery mechanisms, such as injection or encapsulation, require site-specific calibration; and monitoring and validation protocols are essential for ensuring consistent performance. Regulatory approval and public acceptance remain barriers in some regions, particularly for engineered strains.

## 4. Challenges and Limitations

### 4.1. Biological Variability and Environmental Sensitivity

Bacterial systems are inherently sensitive to environmental conditions, including pH, temperature, moisture, salinity, and substrate availability. In the context of construction biotechnology, this variability can lead to unpredictable outcomes in bacterial performance, particularly under fluctuating field conditions [116]. For instance, the efficacy of MICP may vary based on soil mineralogy and the presence of competing ions, making consistency a significant engineering concern [117]. Additionally, the genetic drift of bacterial strains and their response to stressors may lead to metabolic instability [118]. To address this, synthetic biology and bacterial consortia engineering are being explored to enhance robustness and control [119].

### 4.2. Longevity and Durability in Real-World Conditions

The long-term viability of microorganisms embedded in concrete or soil systems remains unclear. In highly alkaline environments such as concrete (pH ~12–13), bacterial spores may survive but have reduced activity over time [120]. Field studies have revealed a gradual decline in metabolic output, particularly for self-healing applications, raising concerns about the sustainability of bacterial effects beyond a few years [46]. Emerging research into smart carriers, nutrient minimum, and dormant-state activation mechanisms aims to mitigate this challenge by prolonging bacterial activity in built environments [121].

Recent studies have increasingly addressed the long-term performance and durability of biotechnological materials under environmental and mechanical stress. For example, bio-consolidated mortars produced through *Bacillus*-mediated carbonate precipitation have demonstrated a significant increase in compressive strength after 180 days during wet–dry cycles [112]. Similarly, biopolymer-enhanced concretes exhibit improved flexibility and crack-bridging capabilities, which contribute to enhanced self-healing performance over time [122]. In general, many bio-based systems show promising short-term mechanical and ecological performance, but concerns remain about their ageing behaviour in real-world conditions.

### 4.3. Regulatory and Safety Considerations

The use of live bacteria in civil engineering introduces a complex layer of biosafety and environmental regulation. Concerns include the potential release of genetically modified organisms (GMOs), horizontal gene transfer in soil ecosystems, and occupational health risks for workers [109]. Currently, there is no unified regulatory framework for bacterial construction applications, and guidelines vary significantly by region. Standardisation is critical for facilitating commercial adoption. Proposals include certification of bacterial strains, ecological risk assessments, and pilot-scale validation for safety [123].

### 4.4. Economic Feasibility and Scalability

While bacterial techniques show promise in terms of environmental and functional applications, their large-scale economic viability remains uncertain. High costs associated with bacterial cultivation, nutrient delivery, and carrier materials often make them less competitive than conventional alternatives, especially in price-sensitive markets [77]. Additionally, process scalability—from lab to field—faces hurdles in yield optimisation, equipment compatibility, and quality control [124]. Cost-benefit analyses are now being developed to evaluate bacterial systems not just in terms of direct costs, but also in terms of lifecycle savings, carbon offsets, and resilience dividends.

### 4.5. Biosafety

Biosafety is a fundamental concern in the deployment of bacterial systems for construction applications. While many of the strains employed in construction biotechnology, such as *S. pasteurii*, *B. subtilis*, and *P. stutzeri*, are generally regarded as safe (GRAS), the introduction of live microorganisms into the built environment raises important ecological, occupational, and public health considerations. The uncontained release of bacteria, particularly genetically modified organisms (GMOs), into soils, water systems, or air necessitates careful ecological risk assessments. However, their long-term viability, potential for ecological drift, and interaction with native microbial communities warrant ongoing monitoring. Key concerns include unintended gene transfer to native microbiota, disruption of local microbial ecosystems, and potential alteration of soil biochemical cycles. These risks are exacerbated in large-scale or long-term applications, where bacterial persistence and interaction with environmental factors are less predictable. Current mitigation strategies involve using non-replicative or non-pathogenic strains, spore-forming bacteria, and biocatalysts that do not contain live cells to enhance environmental safety [9].

Construction sites involve close human interaction with materials, necessitating rigorous screening of bacterial agents for pathogenicity, allergenicity, and toxigenicity. Even non-pathogenic strains may elicit immunological responses or cause dermal irritation, particularly in sensitive populations. Therefore, standardised biosafety protocols are essential, including strain certification, protective handling procedures, and site-specific health risk assessments [10]. One of the primary challenges is the absence of harmonised international regulatory frameworks for bacterial construction applications. Current regulations vary significantly across countries, particularly regarding GMOs. To facilitate safe commercial adoption, a unified global guideline should address criteria such as strain origin, genetic modifications, environmental release, and biosafety testing. Proposed frameworks emphasise ecological impact assessments, monitoring protocols, and stakeholder responsibility in ensuring biosafety compliance [9,10]. To support responsible innovation, collaboration between microbiologists, engineers, policymakers, and public health experts is essential. Educating the public about the safety profiles and environmental benefits of bacterial technologies will help foster acceptance. Biosafety must not be viewed as a constraint but as an enabling foundation for the ethical advancement of living infrastructure technologies [11]. Microbial viability decreases significantly within weeks to months following application, particularly in environments with fluctuating pH, temperature, and humidity. For example, encapsulated bacterial spores used in self-healing concrete may survive for several years under dry conditions but lose activity if exposed prematurely to moisture or oxygen [125]. This natural decline in activity is generally viewed as beneficial from a biosafety standpoint, as it limits the uncontrolled proliferation of applied strains. Moreover, degradation byproducts of microbial processes are typically inert and non-toxic. Nonetheless, comparative long-term studies are still limited, and regulatory frameworks for large-scale use of living materials in construction are evolving. As such, future work should integrate biosafety assessments into performance evaluations, ensuring that the adoption of biological technologies in civil engineering is both sustainable and safe over their whole lifecycle.

## 5. Future Outlook

### 5.1. Synthetic Biology and Engineered Bacteria

Synthetic biology offers unprecedented precision in designing bacterial functionalities tailored for civil engineering. Through the modulation of gene circuits, bacteria can be programmed to initiate carbonate precipitation, form biofilms, or sequester pollutants under specific environmental triggers [104]. Engineered Living Materials are a prominent example, where bacteria are embedded into materials that respond adaptively to external conditions, exhibiting self-repair, self-cleaning, or bio-sensing properties [126]. Future developments aim to build bacterial consortia that perform multi-step biochemical tasks in dynamic environments, thereby moving beyond single-species solutions. These biologically intelligent systems could revolutionise how infrastructure is conceptualised, shifting from static assemblies to responsive, living systems [127].

### 5.2. Digital Tools and Modelling in Bioconstruction

Computational tools are playing a pivotal role in modelling microbe-material interactions, growth kinetics, and performance outcomes. Digital twin frameworks are being developed to simulate bacterial behaviour in engineered systems, enabling predictive optimisation of self-healing concrete or biogrouted soils [128]. Generative design tools also integrate bacterial growth patterns into the early stages of architectural design. The convergence of Building Information Modelling (BIM), bacterial system modelling, and AI is fostering a new era of “bio-integrated design,” enabling architects and engineers to co-design living infrastructure components with real-time feedback loops [129,130].

### 5.3. Integration into Green Building Certifications

As sustainability standards evolve, the integration of bacterial technologies into green certifications, such as Leadership in Energy and Environmental Design (LEED), Building Research Establishment Environmental Assessment Method (BREEAM), and the Living Building Challenge, is becoming increasingly relevant. The described processes related to construction biotechnology improve material circularity but also align with decarbonisation goals for the built environment. For bacterial methods to gain mainstream certification, frameworks must be expanded to include performance-based criteria for bio-based systems, lifecycle assessments, and bacterial ecological impact indices. Institutions such as the International Living Future Institute have already begun evaluating biogenic materials for integration into green ratings [131].

### 5.4. Cross-Disciplinary Research Initiatives

The future of construction biotechnology lies in cross-disciplinary collaboration. Integrating knowledge from microbiology, materials science, computational design, regulatory studies, and architecture is crucial for the scalable and responsible deployment [132]. Programmes like BioMateriOME, NILL 1.0, and SynBioBuild represent pioneering initiatives where biological engineers and built environment experts collaborate to co-create bacterial-enabled construction solutions [133]. International education and public–private consortia are expected to drive innovation, standardisation, and training across regions, ensuring that bacterial engineering transitions from a niche innovation to a structural paradigm in future infrastructure [134].

## 6. Conclusions

Bacterial construction technologies, particularly those utilising MICP and biopolymers, present a transformative opportunity for sustainable infrastructure development. This review has highlighted the breadth of bacterial applications—from crack healing and biomineralisation to soil stabilisation and steel surface protection—demonstrating their potential to reduce environmental impact, improve material durability, and potentially contribute to carbon-neutral construction practices. Despite significant laboratory successes, several challenges hinder large-scale adoption. These include variability in bacterial activity under field conditions, uncertainties in long-term durability, and the absence of standardised regulatory and biosafety protocols. The integration of extremophiles and genetically optimised strains offers promising solutions to enhance performance in diverse environmental settings. To accelerate industrial deployment, interdisciplinary collaboration is essential. Future research should focus on scale-up trials, cost optimisation, environmental risk assessment, and the development of robust regulatory frameworks. Public awareness and acceptance will also be critical in facilitating the transition of these technologies from experimental prototypes to mainstream engineering solutions. In conclusion, bacterial-based construction represents a paradigm shift in civil engineering, offering nature-inspired, resource-efficient, and adaptive alternatives to conventional practices. With continued innovation and systemic integration, these biotechnologies hold the potential to redefine the future of sustainable infrastructure.

## Figures and Tables

**Table 1:** Comparative overview of central bacterial carbonate precipitation mechanisms used in construction biotechnology.

Mechanism	Bacterial Action	Applications	Key Bacteria	Environmental Considerations	Related Ref.
Ureolysis-induced carbonate precipitation	Ureolytic bacteria hydrolyse urea → carbonate → CaCO_3_	Soil stabilisation self-healing concrete surface hardening crack repair corrosion inhibition	*S. pasteurii*	increase in pH value ammonia toxicity porous media distribution	[28,39,42]
Denitrification-induced carbonate Precipitation	Facultative anaerobic bacteria reduce nitrate to N_2_ and carbonate	Anaerobic soil zones deep foundations ureolysis alternative	*P. denitrificans*, *Paracoccus* spp.	Anaerobic, nitrate-rich zones	[38,53]
Sulphate Reduction and Biogenic Metal Precipitation	Sulphate-reducing bacteria → sulphide → metal sulphides	Heavy metal immobilisation corrosion control industrial remediation	*Desulfovibrio* spp.	Anaerobic, sulphur-rich environments	[53]
Photosynthetic Carbonate Precipitation	Cyanobacteria photosynthesis ↑; pH → carbonate precipitation	Living building skins CO_2_ sequestration light-exposed panels	*Synechococcus*, *Gloeocapsa*, *Spirulina*	Light-exposed, alkaline surfaces	[54]

**Table 2:** Comparison of bioconsolidation, biocementation, and bioaggregation.

MICP-Related Technique	Bioconsolidation	Biocementation	Bioaggregation/Biogrounting
Definition	Bacterial-induced hardening of porous substrates via in situ mineral precipitation	Binding of granular particles via bacterial mineral precipitation at contact points	Particle cohesion via secretion of extracellular polymeric substances (EPS)
Mechanism	Filling of pores and voids with bacterially precipitated minerals	Targeted CaCO_3_ deposition between soil grains	EPS-mediated flocculation and particle cohesion
Primary Bacterial Activity	MICP	MICP	MICP with (strong) EPS production
Main Materials Involved	Soils, sandstones, concrete, natural stone, heritage materials	Granular soils (sand, silt), cement-treated zones	Clay, silts, organic soils, and sludge
Engineering Applications	Stone conservation, monument repair, crack sealing in cement-based materials, etc.	Soil stabilisation, liquefaction mitigation, ground improvement, self-healing system for cement-based materials, etc.	Slope stabilisation, erosion control, and moisture retention
Key Benefits	Non-invasive, improves durability and permeability	Increased shear strength, stiffness, and erosion resistance	Improves cohesion, water retention, and supports vegetation
Environmental Sensitivity	Moderate (depends on pH, nutrients, and moisture)	High (sensitive to pH, flow, saturation)	Low to moderate (depends on bacterial growth and EPS production)
Typical Bioagent	*Bacillus* spp. *S. pasteurii*	*S. pasteurii* *Pseudomonas* spp.	*Pseudomonas* spp. *Azotobacter* spp., *Bacillus subtilis*
Related References	[55]	[56]	[51,57]

**Table 3:** Biocleaning set-ups in civil engineering practice.

Case Study	Bacteria	Target Material/Deposit	Carrier Medium	Application Method	Remarks	Ref.
Camposanto Monumentale, Italy	*P. stutzeri*	Nitrate efflorescence	Carbogel-based gel	Topical gel layer for 12–24 h	Validated on high-value frescoes; nitrate removal was successful	[58]
Stone church façade, Spain	*Desulfovibrio desulfuricans*	Gypsum/black crust (sulfated pollutants)	Agar-gel matrix	Surface patching with gauze sheets for 60 h	Restoration-grade stone surfaces showed no damage	[62]
Triumph of Death fresco, Italy	*P. stutzeri* A29	Animal glue	Hydrogel in cellulose matrix	Surface patching (biotretment for 3 h; prolonged baterial treatmen (6 h) also tested;	Preliminary trials confirmed efficiency with low residue	[63]
Brick heritage walls, Romania	Halotolerant *Bacillus* spp.	Mixed salt and pollution crusts	Dry gel with moisture activation	Compressed dry pad application for 30 days	Adapted for large-scale outdoor use	[64]

**Table 4:** Key bacterial biopolymers in eco-engineering and construction.

Bacterial Biopolymer	Producing Microorganism	Key Properties	Applications	Ref.
Xanthan gum	*Xanthomonas campestris*	High viscosity, shear-thinning, stable in extreme pH	Soil stabilisation, erosion control, and eco-grouting	[81]
Curdlan	*Agrobacterium* spp.	Forms gels upon heating, with high water retention	Moisture control, sustainable binders	[82]
Alginate	*Pseudomonas* spp. *Azotobacter* spp.	Ion-sensitive gelation, strong water-binding	Crack sealing, biocementing agent	[83]
Dextran	*Leuconostoc* spp.	Cohesive, improves soil aggregation	Soil cohesion enhancement, dust suppression	[84]
Gellan gum	*Sphingomonas elodea*	Thermally stable, forms rigid gels	Admixture in self-healing mortars	[85]

## Data Availability

The original contributions presented in the study are included in the article; further enquiries can be directed to the corresponding authors.
